# 

**Published:** 1887-12-24

**Authors:** 


CONTENTS.

**A Halt in Life's March** - 213

**Words of Consolation.**—"Peace and Goodwill" - 214

**A Christ's Mass in Hospital** - 214

**Scarlet Fever Convalescents.** By the Right Hon. W. E. Gladstone, M.P. - 215

**The Doctor's Pulpit.**—The Christmas Pudding - 216

**Turning Over New Leaves.**—A Jumble of Hospital Horrors 217

**The Students' Column.**—University of London - 218

**Professional Notes.** By Geo. W. Potter, M.D. - 219

**Notes and News.**—Proposed Infectious Diseases Hospital for Chelmsford.—Hospital Help in Kind.—Cheyne Hospital for Incurable Children.—Mr. Gladstone's Fever Article.—Vacancies.—The Coventry and Warwickshire Hospital.—The Lady Strangford Hospital at Port Said.—Conference of Delegates from Board of Guardians, etc., etc. - 220

**Annotations.**—A Doctor and His Hobby.—Whole Meal Bread.—The Influence of Heredity - 223

**East and West.** By Leslie Keith. Chapter XIII.—Alicia Makes Surrender - 224

**A Children's Paradise** - 226

**The Theatres** - vi.

**Amusements with Profit for all Ages.**—Competitions, Puzzles, Children's Corner, etc. - vi.

The average issue of "The Hospital" now exceeds **10,000 COPIES WEEKLY**, and the circulation is steadily increasing week by week.

